# Intestinal TLR4 deletion exacerbates acute pancreatitis through gut microbiota dysbiosis and Paneth cells deficiency

**DOI:** 10.1080/19490976.2022.2112882

**Published:** 2022-08-18

**Authors:** Mei Qi-Xiang, Fu Yang, Huang Ze-Hua, Yin Nuo-Ming, Wang Rui-Long, Xu Bin-Qiang, Fan Jun-Jie, Huang Chun-Lan, Zeng Yue

**Affiliations:** a Shanghai Key Laboratory of Pancreatic Disease, Shanghai JiaoTong University School of Medicine, Shanghai, China; b Department of Gastroenterology, Shanghai General Hospital, Shanghai JiaoTong University School of Medicine, Shanghai, China

**Keywords:** Acute pancreatitis, TLR4, gut microbiota, intestinal epithelium cells

## Abstract

Toll-like receptor 4 (TLR4) has been identified as a potentially promising therapeutic target in acute pancreatitis (AP). However, the role of intestinal TLR4 in AP and AP-associated gut injury remains unclear. This study aimed to explore the relationship between intestinal TLR4 and gut microbiota during AP. A mouse AP model was establish by intraperitoneal injection of L-arginine. Pancreatic injury and intestinal barrier function were evaluated in wild-type and intestinal epithelial TLR4 knockout (TLR4ΔIEC) mice. Gut microbiota was analyzed by 16S rRNA sequencing. Quadruple antibiotics were applied to induce microbiota-depleted mice. Differentially expressed genes in gut were detected by RNA sequencing. *L. reuteri* treatment was carried out in *vivo* and *vitro* study. Compared with wild-type mice, AP and AP-associated gut injury were exacerbated in TLR4ΔIEC mice in a gut microbiota-dependent manner. The relative abundance of *Lactobacillus* and number of Paneth cells remarkably decreased in TLR4ΔIEC mice. The KEGG pathway analysis derived from RNA sequencing suggested that genes affected by intestinal TLR4 deletion were related to the activation of nod-like receptor pathway. Furthermore, *L. reuteri* treatment could significantly improve the pancreatic and intestinal injury in TLR4ΔIEC mice through promoting Paneth cells in a NOD2-dependent manner. Loss of intestinal epithelial TLR4 exacerbated pancreatic and intestinal damage during AP, which might be attributed to the gut microbiota dysbiosis especially the exhausted *Lactobacillus. L. reuteri* might maintain intestinal homeostasis and alleviate AP via Paneth cells modulation.

**Abbreviations:** AP Acute pancreatitis, TLR4 Toll-like receptor 4, IL-1β Interleukin-1β, IL-6 Interleukin-6, TNF-α Tumor necrosis factor-α, SIRS Systematic inflammatory response syndrome, LPS Lipopolysaccharides, SPF Specific pathogen-free, ZO-1 Zonula occludens-1, CON Control, H&E Hematoxylin and eosin, FISH Fluorescence in situ hybridization, DAPI 4′,6-diamidino-2-phenylindole, PCoA Principal co-ordinates analysis, SCFA Short chain fatty acid, LEfSe Linear discriminant analysis Effect Size, ANOVA Analysis of variance, F/B Firmicutes/Bacteroidetes, PCA Principal component analysis, NOD2 Nod-like receptor 2, ABX antibiotics, PCNA proliferating cell nuclear antigen

## Introduction

Acute pancreatitis (AP) is one of the most common acute gastrointestinal conditions with increasing incidence and significant healthcare burden.^1^. Gut homeostasis is disturbed during the pathogenesis of AP, which could lead to intestinal bacterial translocation.^2^ The intestinal flora, or products and toxins derived from microorganisms, enter the circulation and lead to sepsis and multi-organ failure, which is one of the main causes of death in AP patients.^3^ Therefore, maintaining intestinal homeostasis may serve as a novel target in the treatment of severe AP.^4^

Toll-like receptors (TLRs) are the key activating receptors of innate immune response and involved in inflammation modulation.^5^ In recent years, TLRs have received great attention in AP. TLRs such as TLR2, TLR4 and TLR9 are thought to be the major receptors for recognizing bacteria and have been found to be up-regulated in AP.^6^ Moreover, TLR4 mediates the recognition of bacterial lipopolysaccharides (LPS) and is thought to be highly correlated with system inflammatory reaction syndrome (SIRS), hence TLR4 has been widely studied as a potential mechanistic target for the treatment of AP.^6^ However, TLR4-related researches in AP were controversial and the methods using for previous studies all focused on systemic rather than local knockout of this receptor.^7,8^

TLR4 was found to be highly expressed in the intestine during AP.^9^ In healthy states, TLR4 in epithelial cells contributes to the homeostasis of the gut environment by shaping the host microbiota and maintaining the integrity of the intestinal barrier.^10^ As the key component of intestinal ecosystem, intestinal microbiota could protect the gut barrier and mediate the immune and metabolism of the host. The loss of TLRs in intestinal epithelium have been reported to promote acute intestinal infections, metabolic syndrome and other diseases by affecting the intestinal microbiota.^11–13^ However, the effect of altered intestinal TLR4 expression on AP is still unclear. Furthermore, recent studies suggested that there is a strong link between gut microbiota and severity of AP,^14^ while the interaction between TLR4 and the gut microbiota in AP needs further exploration.

The purpose of this study was to explore the effects of intestinal TLR4 on pancreatic inflammation and intestinal functions in AP model of mice. Moreover, we also investigated related changes in gut microbiota and its potential role during AP after intestinal TLR4 deletion.

## Materials and methods

### Mice

All animal researches were approved by the Animal Care and Use Committee of Shanghai Jiao Tong University (SYXK 2013–0050, Shanghai, China). Male C57BL/6 mice (6–8 w, 20–22 g) were obtained from Shanghai SLAC Laboratory Animal Co. Ltd (Shanghai, China) . *TLR4^fl/fl^* mice and Villin^Cre^ mice were purchased from Shanghai Model Organisms Laboratory (Shanghai, China). *TLR4^fl/fl^* mice were bred to Villin^+/Cre^ mice to generate intestines‐specific Villin^+/Cre^/*TLR4^fl/fl^* mice, abbreviated as TLR4ΔIEC. All mice were maintained under specific pathogen free conditions with free access to water and standard rodent diet. They were housed in room temperature at 22°C and 12 h dark/light cycle and were allocated randomly into groups (n = 6 per group).

### Induction of AP and intervention

L-arginine-induced AP model was used in this study. As described in previous research,^15^ mice in the AP group received two intraperitoneal injections of 8% L-arginine (4 g/kg, pH = 7.0) with a 1 h interval between injections. Mice in the control (CON) group received normal saline (NS) injection. In the L-arginine-induced AP model, the second injection was defined as day 0. To assess the intestinal injury during AP, mice were sacrificed at 1, 2, 3, 4 and 5 days after AP induction. To identify the role of intestinal TLR4, the AP model was induced by L-arginine in wild-type (WT) C57BL6/J and intestines‐specific TLR4−/− mice with a C57BL6/J background. Mice were sacrificed at 3 days after initial injection while the most severe intestinal damage was shown at that time point during AP. All mice received gavage of *L. reuteri* (1 × 10^8^ CFU) for 28 days and then were induced AP model with L-arginine.

Two other kinds of murine models of AP were also established in this study. In caerulein-induced AP, mice were injected with caerulein (100 μg/kg, i.p. 10 times with a 1 h interval between injections) and LPS (5 mg/kg) was administered by i.p. injection immediately after the last injection of caerulein. Mice were sacrificed at 12 h after the initial injection of caerulein. In sodium taurocholate-induced AP, mice were infused with 2% sodium taurocholate solution at a volume of 50 μl /20 g via the biliopancreatic duct at the speed of 5 μl/min and sacrificed at 24 h after the initial injection of sodium taurocholate.

In the series of experiments, mice were divided into six groups (n = 6): control (CON), control-TLR4ΔIEC (TKCON), AP, AP-TLR4ΔIEC (TKAP), AP-*Lactobacillus* (AP+LR) and AP-TLR4ΔIEC-*Lactobacillus* (TKAP+LR). The AP model was induced by L-arginine. Mice in the LR group received gavage of *L. reuteri* (1 × 10^8^ CFU) for 28 days. Mice were anesthetized with chloral hydrate and then sacrificed at 3 days after the first injection of L-arginine. The distal ileum, pancreas, and luminal content of cecum were collected.

### Statistics

All the measured data were displayed as means ± SEM and the analysis were performed using GraphPad prism 8.0 software (San Diego, CA). For comparison of two groups, the student *t*-test was used. For comparison of more than two groups, single factor analysis of variance (ANOVA) was performed. Kruskal-Wallis test was applied for data that did not meet the normal distribution. Differences were indicated statistically significant at *p* < .05.

The additional material and methods were provided in the Supplemental information.

## Results

*Intestinal TLR4 knockout aggravates injury of pancreas and ileum in acute pancreatitis*

Homeostasis of the intestines was reported to play a critical role in AP development. Consistent with previous studies, severe intestinal damage has been found during AP in our study. Moreover, the histopathological injury of ileum increased over time, peaked at 3 days after L-arginine injection and then decreased in accordance with the pancreatic damage (Fig S1a-d). Intriguingly, the critical component of intestinal epithelial cells like Paneth cells, goblet cells and stem cells (Lgr5+) also displayed a significant loss (Fig S1c-d). Notably, compared with TLR2 and TLR9, the mRNA and protein expression levels of TLR4 showed the significant increase in pancreas and ileum after AP induction (Fig S2a-d). TLR4 was highly expressed in intestinal epithelium and peaked at 3 days after AP induction similar to the trend of histopathological injury of ileum during AP (Fig S2b,d,e).

**Figure 1.**
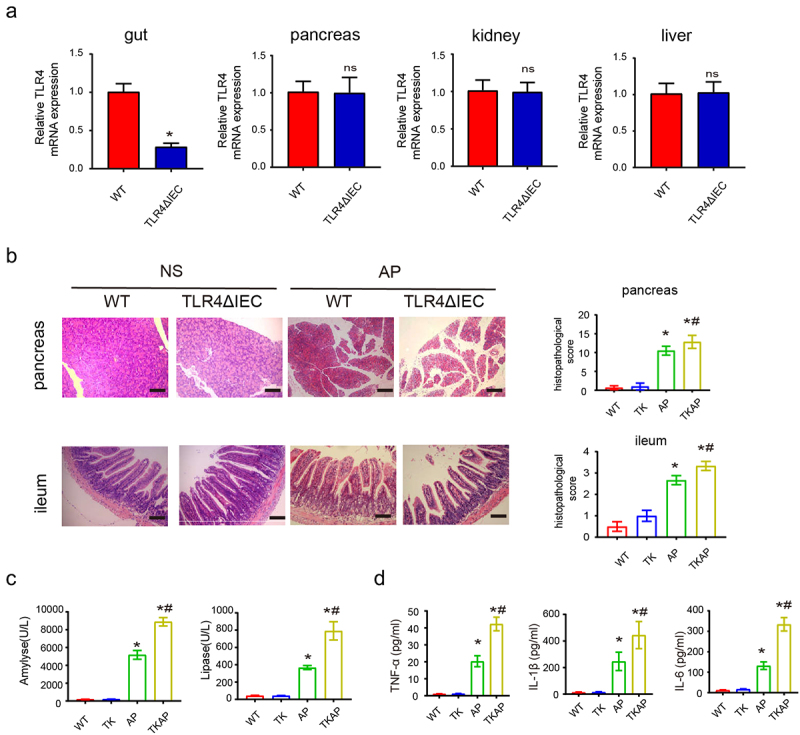
Silencing intestinal TLR4 aggravates injury of pancreas and ileum in acute pancreatitis.

We established TLR4ΔIEC (intestinal specific TLR4 knockout) mice to further determine the role of intestinal TLR4 in AP. Expression mRNA levels of TLR4 in TLR4ΔIEC mice showed a remarkable reduction in the intestine, but not in the pancreas, liver or kidney. These results confirmed that the TLR4ΔIEC mice had intestine-specific knockdown of TLR4 rather than systemic knockdown (Figure 1a). L-arginine-induced AP model was applied to compare the injury and inflammation between WT and TLR4ΔIEC mice during AP. TLR4ΔIEC mice displayed increased ileal and pancreatic injury, serum amyl

(a)mRNA expression of TLR4 of different organs in wild-type mice and TLR4ΔIEC mice. (b) Representative images of pancreas and ileum stained with hematoxylin and eosin. (c) The serum levels of amylase and lipase. (d) The serum level of IL-1β, TNF-a and IL-6. Data are provided as the mean ± SEM (n = 6 per group). *means *p*< .05 *vs* WT, ns means *p*> .05 vs WT, #means p < .05 *vs* AP.

ase, lipase and proinflammatory cytokines (IL1β, IL-6, and TNF-α) compared with WT mice (Figure 1b-d). Furthermore, TLR4ΔIEC mice also displayed exacerbated pancreatic and ileal injury in both caerulein-induced AP model and sodium taurocholate-induced AP model (Fig S3a-b). All these data indicated that silencing intestinal TLR4 aggravated AP.

*Intestinal TLR4 knockout aggravates intestinal barrier injury and bacterial translocation during acute pancreatitis*

**Figure 2.**
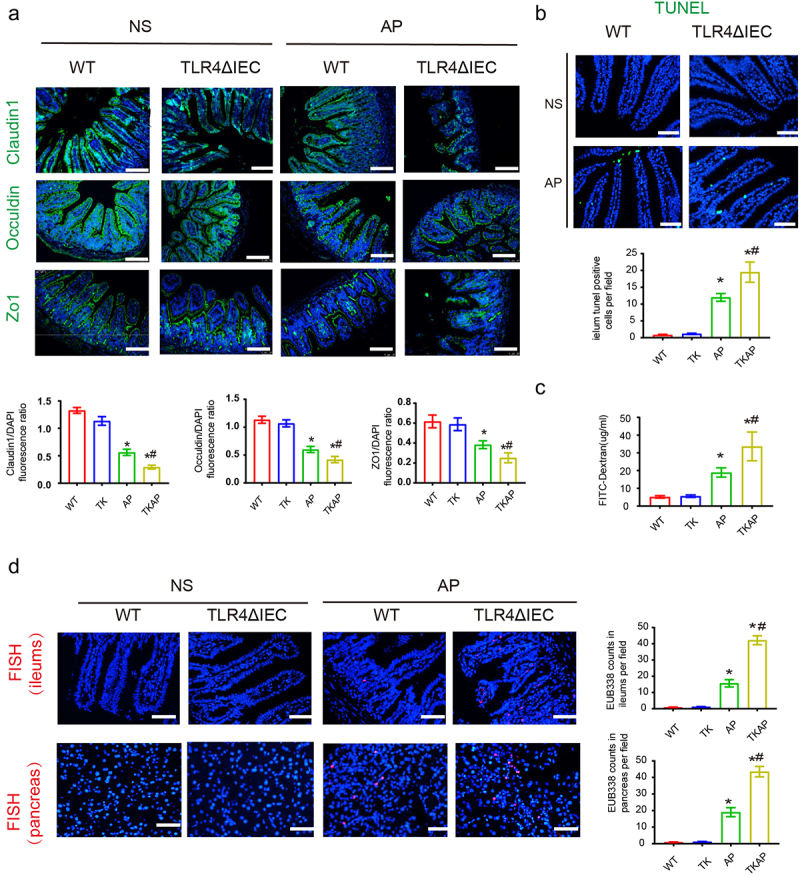
Silencing intestinal TLR4 aggravates intestinal barrier injury and bacterial translocation during acute pancreatitis.

Based on the aggravated damage of ileum and pancreas during AP in TLR4ΔIEC mice, we then investigated the intestinal barrier function and bacterial translocation in WT and TLR4ΔIEC mice. The tight junctions of IECs were assessed by immunofluorescence. Notably, compared with WT mice, the expression levels of Zo-1, Occludin, and Claudin1 were slightly decreased in the TLR4ΔIEC mice. TKAP group showed remarkable decreased expression of intestinal tight junction protein (Zo-1, Occludin and Claudin1) compared with AP group (Figure 2a). Meanwhile, TUNEL staining showed more severe intestinal epithelial apoptosis in TLR4ΔIEC mice than that in WT mice during AP (Figure 2b). FITC assay was used to measure intestinal permeability. Intestinal permeability was significantly increased in AP group. And this adverse result were further exacerbated in TKAP group (Figure 2c). Bacterial translocation in the intestinal epithelium/pancreas was detected by FISH assay using EUB338 probe. AP group exerted increased bacteria translocation in the ileum and pancreas when compared with that in WT group, while this phenomenon in TKAP group was more severe (Figure 2d). These results suggested that TLR4 silenced in IEC promoted bacterial translocation from ileum to pancreas.

(a) Photomicrograph of Occludin, Claudin1 and ZO-1 immunofluorescence in the ileum (100× magnification). The tight junction proteins/DAPI fluorescence ratio per field of view was measured. (b) The apoptosis in the small intestines was assayed by TUNEL. The number of TUNEL-positive cells (green) per field of view was quantified. (200× magnification). (c) FITC assay was used to measure intestinal permeability. (d) The positive hybridizing signal of total bacteria detected by EUB338 probe. EUB338 counts in intestinal epithelium (200× magnification) or pancreas (100× magnification) per field were quantified. Data are provided as the mean ± SEM (n = 6 per group). *means *p*< .05 *vs* WT, #means *p*< .05 *vs* AP.

**Figure 3.**
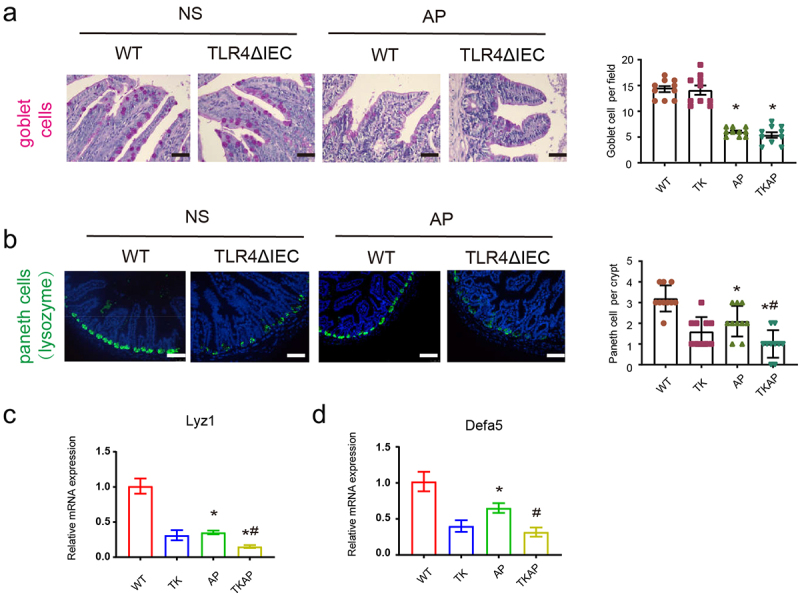
Changes in intestinal cells in TLR4ΔIEC mice during acute pancreatitis.

We then investigated the changes of other intestinal epithelial cells during AP. Goblet cells showed no significant change in WT and TLR4ΔIEC mice (Figure 3a). However, compared with wild type mice, Paneth cells (labeled by lysozyme) showed remarkable reduction in mice of intestinal TLR4 knockout with and without AP (TK and TKAP group) (Figure 3b). Interestingly, The mRNA expression level of Paneth cells-related genes, Lysozyme1 (Lyz1) and α-defensins 5 (Defa5), showed the similar downward trend as Paneth cells. To sum up, our results suggested that TLR4ΔIEC mice exhibited less Paneth cells.

(a) Representative images of intestinal goblet cells stained with PAS (400× magnification). (b) Representative images of intestinal Paneth cells stained with lysozyme by immunofluorescence (200× magnification). (c) Intestinal mRNA expression of Paneth-related genes (Lysozyme1 and Defensin-alpha 5). Data are provided as the mean ± SEM (n = 6 per group). *means *p*< .05 *vs* WT, #means *p*< .05 *vs* AP.

*Microbiota dysbiosis increased in mice of intestinal TLR4 deletion*

**Figure 4.**
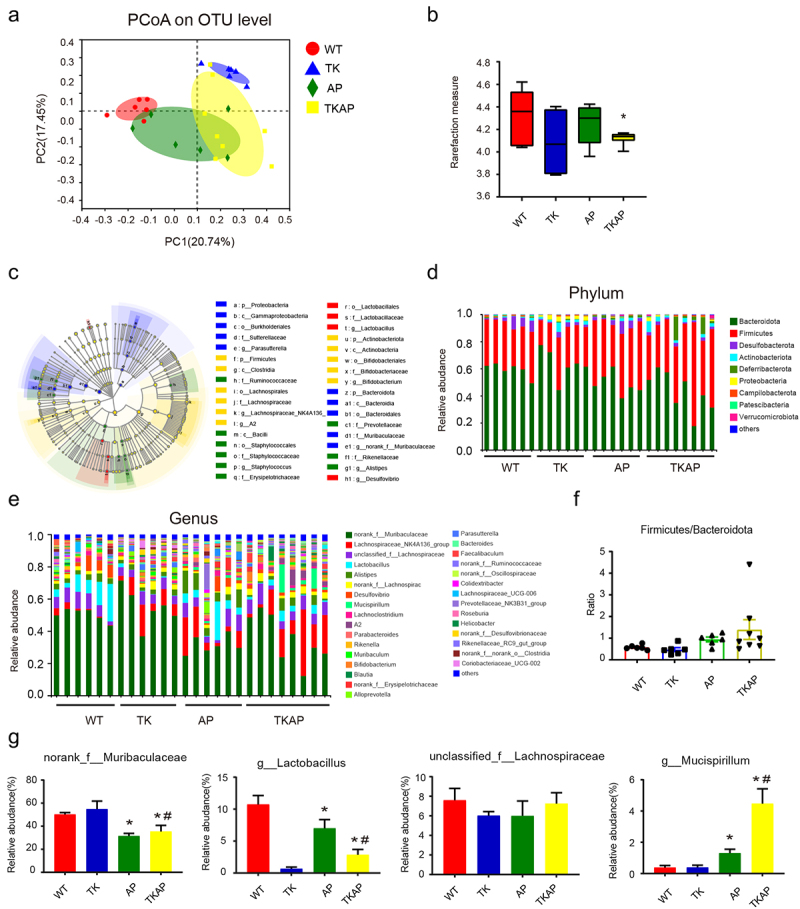
Microbiota dysbiosis was worsened in mice of intestinal TLR4 deletion.

We displayed 16S rRNA sequencing of fecal DNA to investigate the differences in gut microbiota derived from different genotypes. Beta diversity analysis of principal coordinates analysis (PCoA) showed a clear separation of gut microbiota between WT and TLR4ΔIEC mice (Figure 4a). TLR4ΔIEC mice harbored microbiota with lower alpha diversity (demonstrated by Shannon index) than WT mice. This phenomenon was pronounced after AP induction (Figure 4b). The gut microbiota structure at phylum and genus levels was further investigated. TLR4ΔIEC mice exhibited great alteration of gut microbiota (Figure 4c-e). At phylum level, Firmicutes/Bacteroidetes ratio exhibited no significant difference between WT and TLR4ΔIEC mice (Figure 4d). At genus level, the selected most abundant microbiota was shown in Figure 4g. Notably, compared with WT mice, the abundance of *Lactobacillus* remarkably decreased in TLR4ΔIEC mice. The same phenomenon occurs after AP induction (Figure 4g). *Lactobacillus* has been widely studied and reported to be one of the beneficial probiotics with anti-inflammatory effects. The decreased alpha diversity and exhaustion of *Lactobacillus* suggested gut microbiota was disturbed in TLR4ΔIEC mice.

(a) Principle coordination analysis (PCoA) based on OTU abundance. (b) α-diversity analysis between four groups using Shannon index. (c) Cladograms generated by LEfSe showed the differences in taxa among four groups (from phylum to genus levels). (d) The taxonomic composition distribution among four groups on phylum-level of fecal microbiota. (e) The taxonomic composition distribution among four groups on genus-level of fecal microbiota. (f) The ratio of Firmicutes/Bacteroidetes among four groups were shown (phylum-level). (g) Relative abundance of norank_f_*Muribaculaceae*, g_*Lactobacillus*, unclassified_f_*Lachnospiraceae* and g_*Mucispirillum* were shown (genus-level). Data are provided as the mean ± SEM (n = 6 per group). *means *p*< .05 *vs* WT, #means *p*< .05 *vs* AP.

*Gut dysbiosis and decreased Paneth cells are closely related with exacerbation of AP*

**Figure 5.**
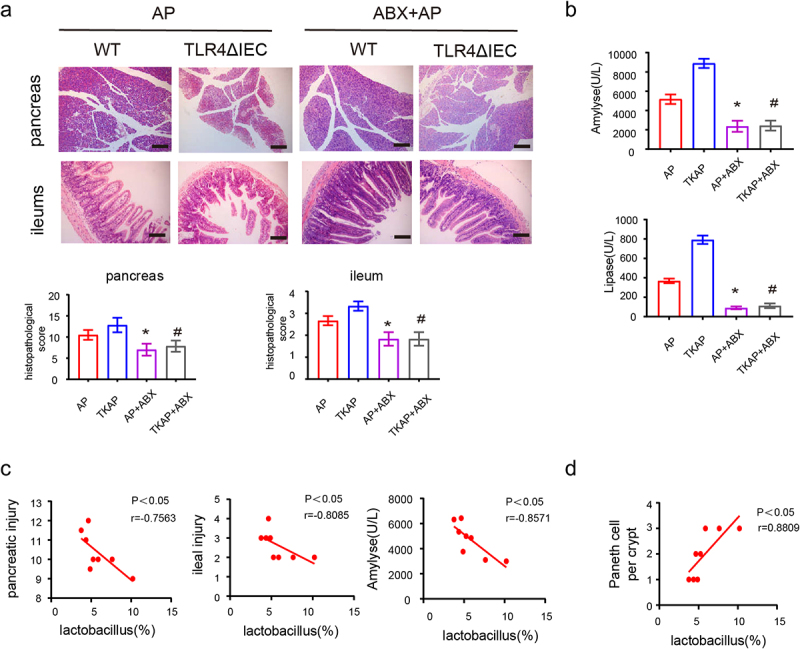
The injury of pancreas and ileum in acute pancreatitis were relieved in mice feed with antibiotics.

To further determine whether the exacerbation of AP in TLR4ΔIEC mice relied on gut microbiota dysbiosis, we pretreated all mice with antibiotics (ABX) for 14 days prior to AP induction to eliminate the gut flora. BHIA plate counting and representative photos of bacterial culture showed that antibiotic treatment depleted mice enteric bacteria (Figure S5a). Concentration of fecal bacterial DNAs and relative fecal 16S bacterial rDNAs before and after antibiotic treatment also confirmed this conclusion (Figure S5b-c). Interestingly, the histology feature of pancreas and ileum, serum amylase and lipase level in WT and TLR4ΔIEC mice model after ABX were indistinguishable (Figure 5a-b).

(a) Representative images of pancreas and ileum stained with hematoxylin and eosin. (b) The serum level of amylase and lipase. (c) Spearman correlation analysis between abundance of *Lactobacillus* and scores of injury (pancreatic injury, intestinal injury and serum amylase). (d) Spearman correlation analysis between *Lactobacillus* and Paneth cells. Data are provided as the mean ± SEM (n = 6 per group). *means *p*< .05 *vs* AP, #means *p*< .05 *vs* TKAP.

In AP, Spearman correlation analysis revealed *Lactobacillus* abundance was negatively correlated with the injury score of pancreas (R^2^ = 0.7563, *p*< .05), the injury score of ileum (R^2^ = 0.8085, *p*< .05) and serum amylase (R^2^ = 0.8571, *p*< .05) (Figure 5c). These results indicated that gut microbiota dysbiosis in TLR4ΔIEC mice represented by lack of *Lactobacillus* are closely related with increased severity of AP .

Notably, we observed that the number of Paneth cells changed in different genotypes in series of AP experiments, which was consistent with *Lactobacillus* abundance changes in these groups (Figure 3b, compared to Figure 4g). Spearman analysis also revealed that *Lactobacillus* abundance was positively correlated with the number of Paneth cells (R^2^ = 0.8809, *p*< .05) (Figure 5d). Our previous study had proved that ablation of Paneth cells by dithizone (Dith) aggravated AP. Collectively, absent *Lactobacillus* and Paneth cells displayed in TLR4ΔIEC mice might play the important role in the exacerbation of AP.

*Microbiota dysbiosis and severity of acute pancreatitis were alleviated in TLR4ΔIEC mice with L. reuteri intervention*

To investigate the critical role of *Lactobacillus* in AP symptoms, WT and TLR4ΔIEC mice received gavage by *L. reuteri* (1 × 10^8^ CFU, 28 days) before AP induction.

**Figure 6.**
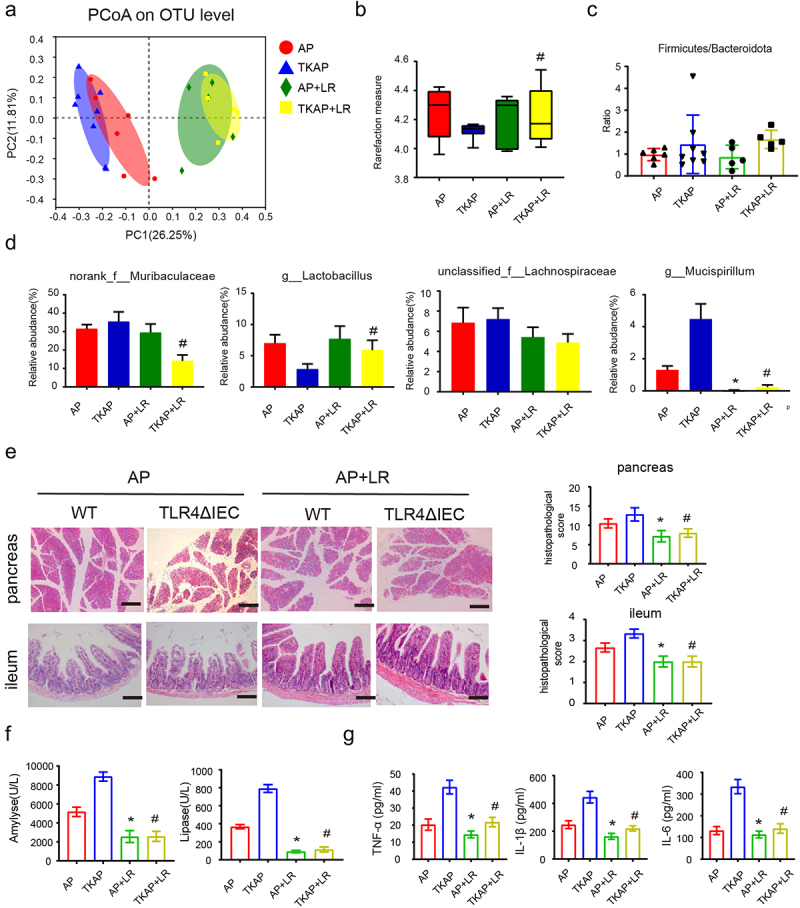
Microbiota dysbiosis and the injury of pancreas and ileum in acute pancreatitis were improved in mice feed with *L. reuteri.*

As shown by 16S rRNA sequencing of fecal DNA, although F/B ratio did not demonstrate significantly change, the decreased alpha diversity and *Lactobacillus* abundance in TLR4ΔIEC mice were recovered after *Lactobacillus reuteri* gavage (Figure 6a-d, Figure S4a-c). These finding indicated that *L. reuteri* feeding improved gut microbiota dysbiosis in TLR4ΔIEC mice.

(a) Principle coordination analysis (PCoA) based on OTU abundance. (b) α-diversity analysis among four groups using (Shannon index). (c) The ratio of Firmicutes/Bacteroidetes among four groups were shown (phylum-level). (d) Relative abundance of norank_f_*Muribaculaceae*, g_*Lactobacillus*, unclassified_f__*Lachnospiraceae* and g_*Mucispirillum* were shown (genus-level). (e) Representative images of pancreas and ileum stained with hematoxylin and eosin (f) The serum levels of amylase and lipase. (g) The serum levels of IL-1β, TNF-a and IL-6. Data are provided as the mean ± SEM (n > 5 per group). *means *p*< .05 *vs* AP, #means *p*< .05 *vs* TKAP.

**Figure 7.**
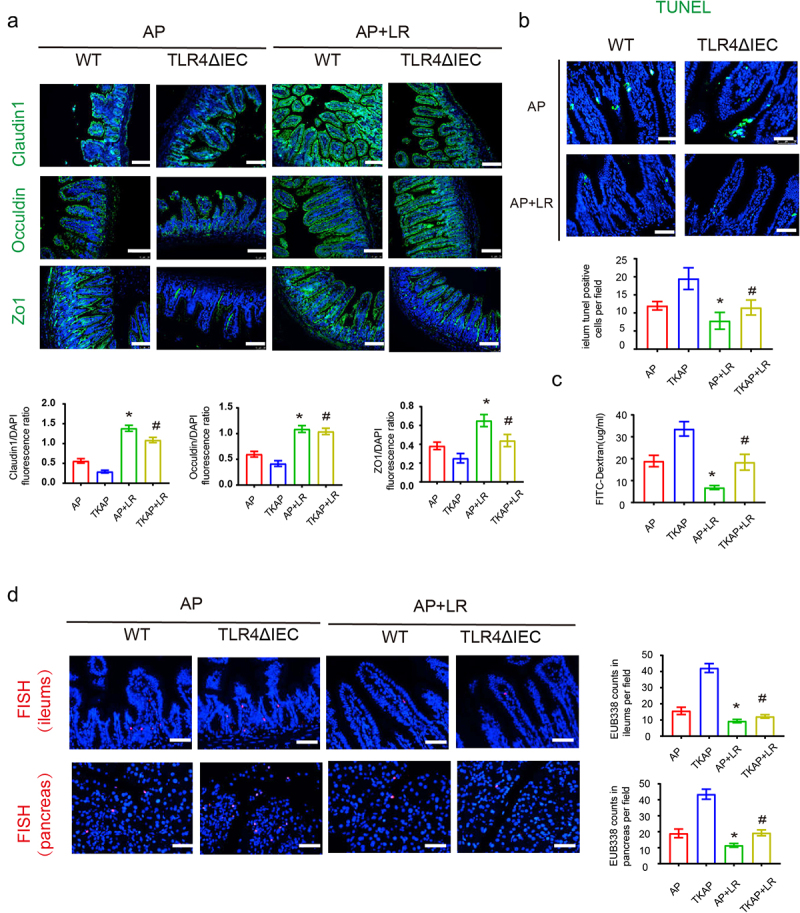
Intestinal barrier injury and bacterial translocation during acute pancreatitis were reduced after feeding *L. reuteri.*

Strikingly, compared with mice incubated with NS, mice in two genotypes treated with *L. reuteri* displayed obviously mitigated AP, including decreased histopathological score, serum amylase and lipase and level of pro-inflammatory cytokines (serum TNF-α, IL-6 and IL-1β) (Figure 6e-g). Furthermore, intestinal barrier injury and bacterial translocation also relieved in *L. reuteri* treated mice. Compare with AP and TKAP group, mice in AP+LR group and TKAP+LR group displayed increased expression level of tight junctions (Zo-1, Occludin and Claudin1) and reduced intestinal epithelial apoptosis, intestinal permeability and bacterial translocation (Figure 7a-d).

(**a**) Photomicrograph of Occludin, Claudin1 and ZO-1 immunofluorescence in the ileum (100× magnification). The tight junction proteins/DAPI fluorescence ratio per field of view was measured. (**b**)The apoptosis in the small intestines was assayed by TUNEL. The number of TUNEL-positive cells (green) per field of view was quantified. (200× magnification). (c) FITC assay was used to measure intestinal permeability. (d)The positive hybridizing signal of total bacteria was detected by EUB338 probe. EUB338 counts in intestinal epithelium (200× magnification) or pancreas (100× magnification) per field were quantified. Data are provided as the mean ± SEM (n = 6 per group). *means *p*< .05 *vs* AP, #means *p*< .05 *vs* TKAP.

To sum up, these data indicated that the administration of *L. reuteri* alleviated pancreatic and intestinal damage in TLR4ΔIEC mice during AP.

*L. reuteri protects mice from AP-induced ileal injury by inducing Paneth cells and maintaining intestinal stem cell number*

**Figure 8.**
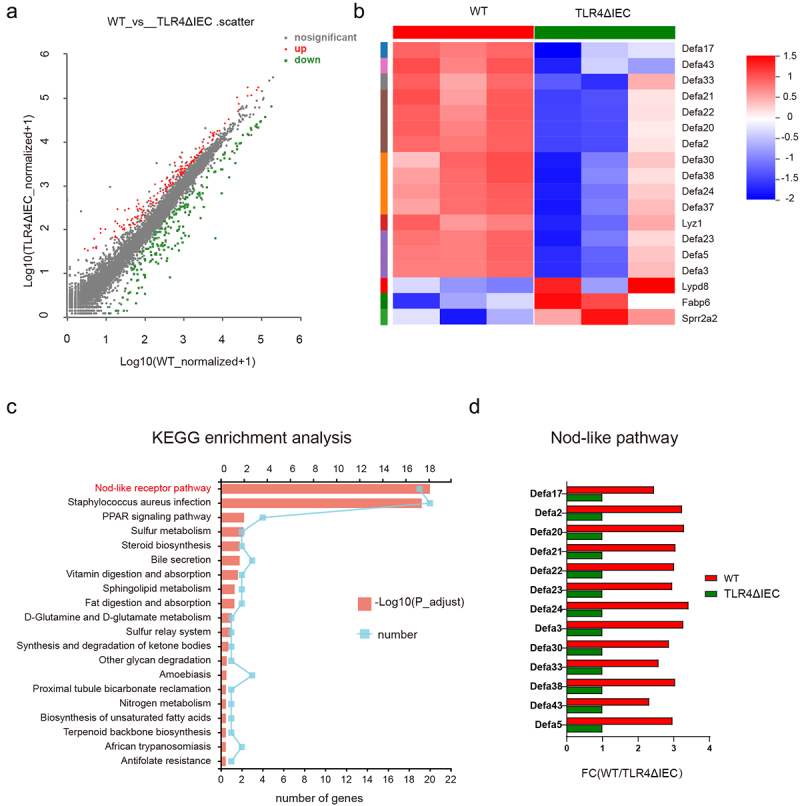
The functions related to Paneth cells were changed after intestinal TLR4 deletion.

RNA sequencing of gut identified 345 differentially expressed genes (161 up, 184 down) between WT and TLR4ΔIEC mice (Figure 8a). Interestingly, as shown in Figure 8b, genes down-regulated by more than two-fold were mostly related to Paneth cells. Additionally, the KEGG pathway analysis also suggested that genes affected by intestinal TLR4 deletion were related to the activation of nod-like receptor pathway (Figure 8c). High enrichment for genes encoding components of the nod-like receptor pathway were shown in Figure 8d. The results confirmed that Paneth cells-related genes were down-regulated in TLR4ΔIEC mice and suggested that the nod-like receptor pathway might be involved in the regulation of Paneth cells.

(a) The Volcano Plot illustrated that there are 345 differentially expressed genes (161 up, 184 down) in gut between WT and TLR4ΔIEC mice (b)The heat map of RNA sequencing showed the expression differences on the microarray in gut between WT and TLR4ΔIEC mice. (c) KEGG pathway analysis indicated down-regulation of the Nod-like receptor pathways in gut of TLR4ΔIEC mice. (d) Fold-change (FC) in genes encoding components of the nod-like receptor pathway in RNA sequencing experiments.

**Figure 9.**
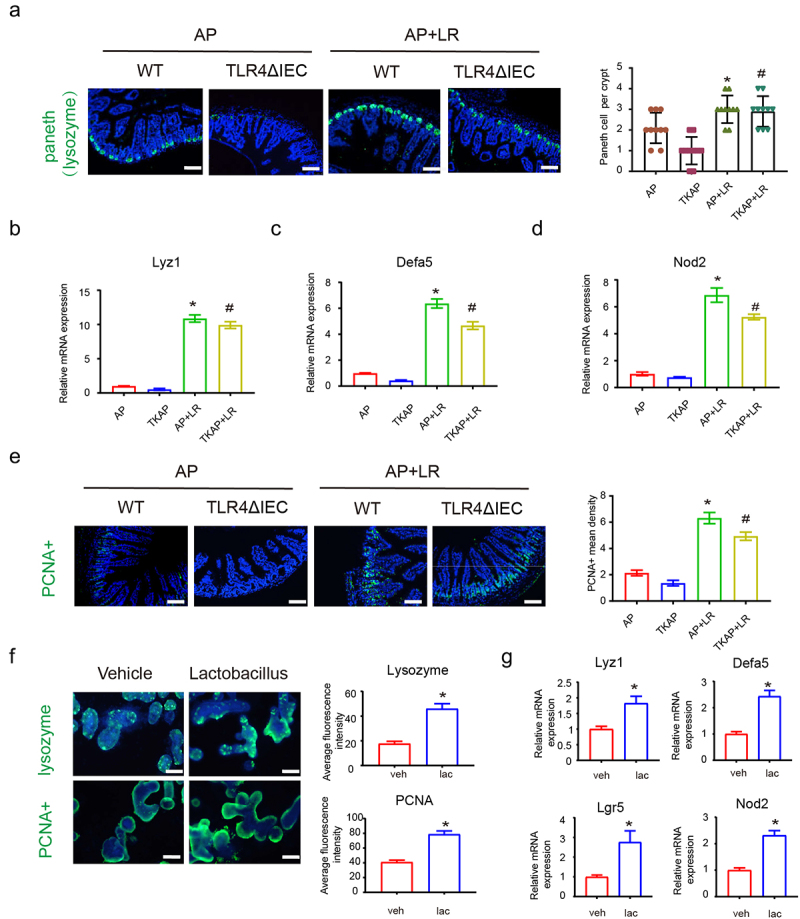
*L. reuteri* protects mice from AP-induced ileal injury by inducing Paneth cells and maintaining intestinal stem cell number.

To further uncover the protective effect of *L. reuteri* against intestines, the function of Paneth cells and stem cells were explored. As shown in Figure 9a-d, administration of *L. reuteri* increased the density of lysozyme+Paneth cells with elevated mRNA expression of antimicrobial peptide genes (Lyz-1 and Defa5) and Nod like receptor pathway genes (Nod2). Additionally, the number of proliferating cell nuclear antigen (PCNA)-positive cells were remarkably increased in the intestines of *L. reuteri* treated mice (Figure 9e). The results indicated that Paneth cells and its related products play the important role in the protective effect of *L. reuteri* during AP.

(a) Representative images of intestinal Paneth cells stained with lysozyme by immunofluorescence (200× magnification). (b-d) Intestinal mRNA expression of Lyz1, defa 5 and Nod2 in mice gut. (e) Representative images of intestinal stem cells stained with PCNA by immunofluorescence (200× magnification). *means *p*< .05 *vs* AP, #means *p*< .05 *vs* TKAP. (f) Representative images of Paneth cells (lysozyme+) and stem cells (PCNA+) in enteroids by immunofluorescence (400× magnification). (g) mRNA expression of Lyz1, defa 5, Lgr5 and Nod2 in enteroids with or without *Lactobacillus* incubation. Data are provided as the mean ± SEM (n = 6 per group). *means *p*< .05 *vs* Vehicle.

To verify that *Lactobacillus* worked via directly promoting Paneth cells rather than regulating the flora, the mice were pretreated with ABX for 14 days to eliminate the gut flora and then injected with 40 mg/kg dithizone every three days for two weeks to ablate Paneth cells before AP induction. Consistent with our previous research, the injury of pancreas and ileum in AP were aggravated after Paneth deletion (Fig S6a-b). However, this phenomenon did not alleviated with simultaneous supplementation of *L. reuteri* (1 × 10^8^ CFU, 2 weeks) (Fig S6a-b).

Then, we used the enteroid model to explore the interaction of IEC and *Lactobacillus*. Enteroids were pretreated with or without *L. reuteri* (1 × 10^6^ CFU) Matrigel for 48 h and then treated with TNF (60 ng/ml) for 12 h to induce intestinal damage to the enteroids. Consistent with our findings *in vivo*, incubation of *L. reuteri* activated the Paneth cells and promoted the proliferation in enteroids. The intensity of lysozyme and PCNA detected by immunofluorescence were increased after administration of *L. reuteri* (Figure 9f). As shown by QPCR analysis (Figure 9g), l. *reuteri* triggered the expression of antimicrobial peptide genes (Lyz1 and Defa5), stem cells-related gene (Lgr5) and Nod like pathway gene (Nod2). The results of our *in vivo* and *in vitro* studies indicated that *Lactobacillus* promoted the function of Paneth cells and stem cells and up-regulated the Nod2 pathway in TLR4ΔIEC mice.

## Discussion

Increasing data suggest a key role of TLR4 in experimental AP. Although some studies have reported that systemic knockdown of TLR4 reduces inflammation in AP models,^16,17^ others have found that this is not significant.^7,8^ Despite the controversy, TLR4 has been regarded as a potentially promising therapeutic target in AP.^6^ However, previous studies mainly focused on the effects of systemic TLR4 knockout during AP, while the role of intestinal TLR4 in AP and AP-associated gut injury remains unclear.

Given the previous finding that TLR4 systemic knockout in mice can ameliorate inflammation of pancreas and lung,^17^ we hypothesized that silencing intestinal TLR4 gene would have attenuated intestinal injury and alleviated AP. Surprisingly, our study revealed that mice deficient in intestinal TLR4 had exacerbated pancreatic and intestinal injury during AP. An explanation for this phenomenon may reside in where different genes work. As an important receptor for inflammation, systemic knockdown of TLR4 in AP models may primarily affect the Toll-like receptor 4 (TLR4) expression on pancreas and macrophages, which plays an important role in the development of AP.^17,18^ However, TLRs are also widely expressed in intestinal epithelial lineages and are involved in the establishment of intestinal homeostasis.^5^ Loss of epithelial TLRs were reported to promote the occurrence of metabolic syndrome by affecting gut microbiota.^12,13^ Dysbiosis of the gut microbiota has been reported to be associated with the severity of AP.^19^ In this study, the indistinguishable phenotype of AP between WT and TLR4ΔIEC mice after antibiotic intervention enabled us to hypothesize that deletion of intestinal TLR4 might exacerbate AP by affecting gut microbiota and intestinal homeostasis. Therefore, we suggested that intestinal TLR4 might serve a protective role in intestinal homeostasis and prevents mice from pancreatic injury rather than exacerbate it.

Similar to previous studies, our findings confirmed disruption of intestinal homeostasis during AP.^2^ The specific manifestations of AP-associated intestinal damage in our study were the decreased number of intestinal epithelial cells, including Paneth cells, goblet cells and stem cells. Notably, the number of Paneth cells was further reduced in intestinal TLR4 deletion mice.

TLRs are widely expressed in intestinal epithelial lineages and are involved in the establishment of intestinal homeostasis. Meanwhile, loss of epithelial TLRs may also lead to malnutrition and increase susceptibility to enteritis and tumor.^6,20^ Activation of epithelial TLR signals can increase the integrity of the intestinal epithelial barrier and enhance tolerance to intestinal flora.^5,10,13^ Abnormal signal transduction of TLR can inhibit the clearance of pathogen, thus promoting the disorder of gut microbiota and reducing bacterial diversity. It has been reported that lacking of TLR4 and TLR5 in intestinal epithelium can promote the occurrence of metabolic syndrome by affecting gut microbiota.^12,13,21^ Dysbiosis of the gut microbiota is believed to be associated with the severity of AP.^19^ In this study, the indistinguishable demonstration of AP between WT and TLR4ΔIEC mice after antibiotic intervention enabled us to hypothesize that deletion of intestinal TLR4 may exacerbate AP by affecting gut microbiota.

Aberrant TLR activation might contribute to dysbiosis via the release of antimicrobial peptides, ROS and RNS. Given that TLR signaling in IECs induced the expression of iNOS and NADPH oxidases resulting in producing nitric oxide and ROS, the growth of facultative anaerobes was affected leading to dysbiosis.^20^ *Lactobacillus* is one of the facultative anaerobes that is naturally found in a variety of hosts and environments, including the healthy human intestine.^22^ Compared with WT mice, the abundance of probiotic like *Lactobacillus* was found remarkably decreased in TLR4ΔIEC mice in our study. *Lactobacillus* has been widely studied and reported to be one of the beneficial probiotics with anti-inflammatory effects.^23^ Down-regulation of *Lactobacillus* has been found to be closely related to irritable bowel syndrome (IBS), inflammatory bowel disease (IBD), type I diabetes, colon cancer, etc.^24–27^ There are several studies focused on *Lactobacillus* and AP. Supplementation of *Lactobacillus* has been found to reduce pancreatic sepsis and the number of surgical interventions in AP patients.^28^ Although some clinical trials have raised concerns about the effectiveness of probiotics, recent studies pointed out that AP patients treated with synbiotics did not have a worse clinical outcome and had lower risk of organ failure and reduced duration of hospital stay.^29,30^ Spearman correlation analysis in our study suggested that absence of *Lactobacillus* and exacerbated AP are closely related.

*L. reuteri* has a profound regulatory effect on host microbiota and immune responses with few safety concerns. Therefore *L. reuteri* is a good candidate for disease prevention and treatment.^23^ *L. reuteri* has demonstrated its therapeutic potential in a variety of diseases such as lupus erythematosus, obesity and intestinal infections.^31–33^ However, its role in AP has not been studied yet. In order to further confirm the core role of *Lactobacillus* in influencing AP, we fed WT and TLR4ΔIEC mice with *L. reuteri*. We observed that feeding TLR4ΔIEC mice *L. reuteri* not only supplemented its lacked *Lactobacillus*, but also restored the decreased bacterial diversity. Strikingly, *L. reuteri* significantly reduced pancreatic and intestinal damage during AP. *L. reuteri* has been extensively shown to have the ability to upregulate the expression of tight junction proteins (ZO-1, Occuldin, Claudin1). D. Ahl et al found that expression of the tight junction proteins were significantly increased in the bottom of the colonic crypts by *L. reuteri* in DSS-induced colitis. Yi et al reported that *L. reuteri* improved expression of tight junction proteins via the MLCK pathway in IPEC-1 cells during challenge with ETEC K88.^34^ Zhou et al reported that *L. reuteri* improved the expression of intestinal tight junction proteins and maintained the integrity of the intestinal barrier by inhibiting apoptosis of intestinal epithelial cells.^35^ Consistent with previous researches, our study showed that *L. reuteri* reduced apoptosis of intestinal epithelium, up-regulated expression level of tight junction proteins, decreased bacterial translocation and ultimately reversed the aggravated pancreatic and ileal injury in TLR4ΔIEC mice. These phenomena suggested that absence of intestinal TLR4 might affect AP via *Lactobacillus*.

Paneth cells are secretory cells in the epithelium of the small intestine and play an essential role in the maintenance of immune homeostasis.^36^ Our previous study revealed that ablation of Paneth cells aggravates AP.^37^ Strikingly, in this study, we also observed the deficiency of Paneth cells in TLR4ΔIEC mice. RNA sequencing of TLR4ΔIEC mice confirmed that Paneth cells-related genes were also down-regulated. These findings indicated that the undesired demonstration in TLR4ΔIEC mice partly attributed to the dysfunction of Paneth cells.

It has been reported that Paneth cells can sense microbial cues via TLR-Myd88 mediated pathways, in turn mounting antimicrobial defense by releasing antimicrobial peptides, lysozyme and phospholipase A.^38^ Our results demonstrated that the microbiota structure was altered in TLR4ΔIEC mice, specifically the absence of *Lactobacillus. L. reuteri* was reported to be effective in promoting the number and function of Paneth cells to inhibit C. rodentium colonization.^39^ Similarly, Spearman correlation analysis in our study revealed that *Lactobacillus* abundance was positively correlated with the number of Paneth cells. Moreover, the deficiency of Paneth cells in TLR4ΔIEC mice was restored after *L. reuteri* feeding. These results suggested that deletion of intestinal TLR4 may affect the number and function of Paneth cells by affecting abundance of *Lactobacillus*.

Interestingly, the KEGG pathway analysis also suggested that genes affected by intestinal TLR4 deletion were related to the activation of Nod-like receptor pathway. NOD2 is a part of the Nod-like receptors, which is highly expressed in ileal Paneth cells and plays a critical role in its antibacterial function.^40^ It is reported that some *Lactobacilli* protect mice from colitis in a NOD2-dependent manner.^41^Our study also observed that NOD2 was induced by treatment of *L. reuteri* in our *in vivo* experiments.

Intestinal organoids techniques have become a powerful tool for studying the interaction between microbiota and the intestinal mucosal barrier.^42,43^ To further explore the intestinal epithelium-*lactobacillus* interactions, enteroids were applied in our study *in vitro*. Paneth cells of small intestine are located at the base of intestinal crypts, intercalated among the active intestinal stem cells.^44^ The new role for Paneth cells has been demonstrated lately in the realm of epithelial regeneration after damage in recent evidence.^45^ It has been reported that Paneth cells not only release Stem cell growth factors, but also have the potential to differentiate into Stem cells, which aids in epithelial restitution.^38^ Consistent with our *in vivo* findings, *L. reuteri* increased the number of Paneth cells and PCNA-positive cells, as well as the expression of antimicrobial peptides in enteroids, indicated that *L. reuteri* activated the Paneth cells and promoted epithelial proliferation. Collectively, these results indicated that in TLR4ΔIEC mice, *Lactobacillus* protects mice from AP-associated gut injury through Paneth cells promotion in a NOD2-dependent manner. However, the effective products of *L. reuteri* that play the major role in promoting Paneth cells during AP still deserve further study.

## Conclusion

The deletion of TLR4 in the intestinal epithelium exacerbates intestinal and pancreatic injury during AP, which may be attributed to dysbiosis of gut microbiota (exhaustion of *Lactobacillus*) and dysfunction of Paneth cells. *L. reuteri* has the ability to modulate Paneth cells and intestinal stem cell proliferation to maintain intestinal homeostasis and alleviate AP. Together, our findings highlight the gut-pancreas axis during AP and provide comprehensive information on the interaction between gut microbiota and intestinal epithelial cells. Furthermore, the supplementation of *Lactobacillus* and Paneth cell-oriented treatments might be the promising therapeutic strategy against AP. Probiotic therapy that contains *L. reuteri* may be the useful and cost-effective approach to ameliorate AP.

## Supplementary Material

Supplemental Material

## Data Availability

The raw data that support the findings of this study are openly available in the SRA database with reference number PRJNA824019(https://www.ncbi.nlm.nih.gov/bioproject/PRJNA824019).
