# Supplementary material for: Intestinal TLR4 deletion exacerbates acute pancreatitis through gut microbiota dysbiosis and Paneth cells deficiency
Source: Gut Microbes. 2022 Aug 18;14(1):2112882. doi: 10.1080/19490976.2022.2112882 (PMC9397436; doi:10.1080/19490976.2022.2112882)
Supplement: Supplemental Material [file KGMI_A_2112882_SM2628.zip › revised supplemental information202208.docx]

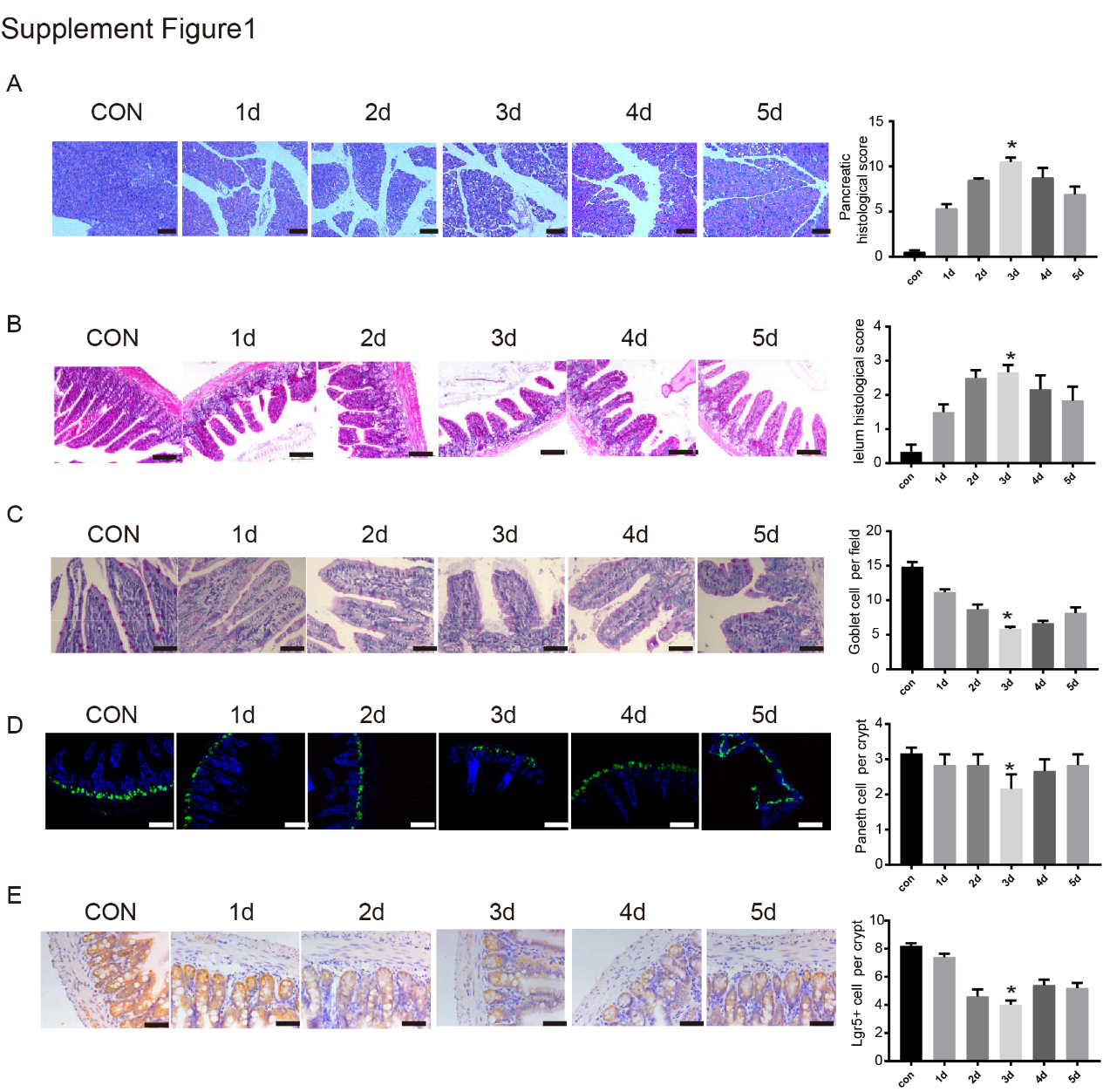


**Supplementary Figure1 Intestinal epithelial cells were damaged in L-arg induced acute pancreatitis in mice**

(a) Representative images of pancreas stained with hematoxylin and eosin (original magnification ×100) (from 0-5d). (b) Representative images of ileum stained with hematoxylin and eosin (original magnification ×200) (from 0-5d). (c) Representative images of intestinal goblet cells stained with PAS (original magnification ×200). (d) Representative images of intestinal paneth cells stained with Lysozyme by immunofluorescence (original magnification ×200). (e) The number of LGR5 + positive cells in intestinal tract of mice was detected by immunohistochemistry (original magnification ×200). Data are provided as the mean ±SEM (n=6 per group). *means p<0.05 vs CON.


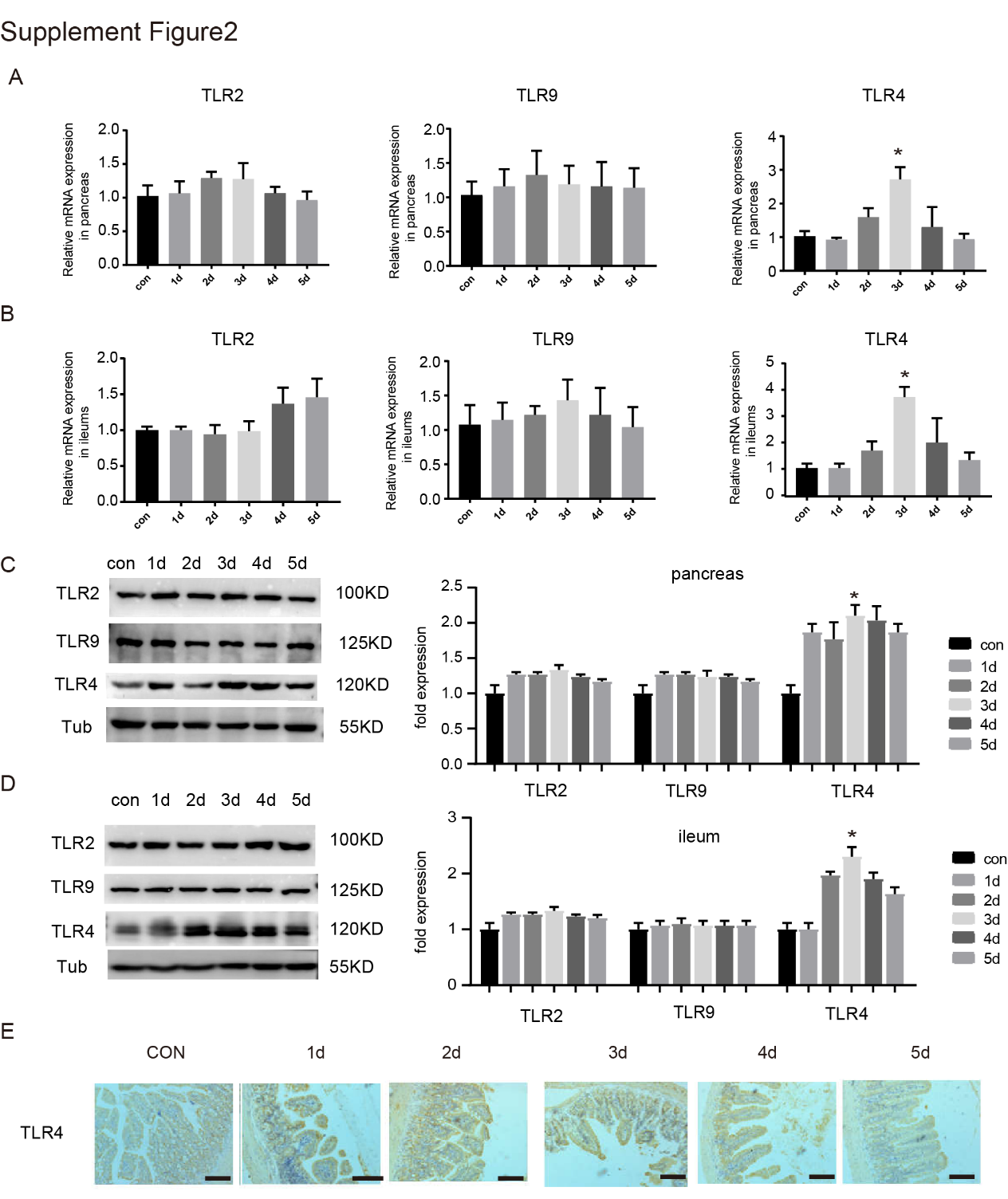


**Supplementary Figure2 TLR2, TLR4 and TLR9 expression in L-arg induced acute pancreatitis in mice**

(**a**) Pancreatic mRNA expression of TLR2, TLR4 and TLR9. (**b**) Intestinal mRNA expression of TLR2, TLR4 and TLR9. (**c**) Pancreatic protein expression of TLR2, TLR4 and TLR9. (**d**) Intestinal protein expression of TLR2, TLR4 and TLR9. (**e**) Representative images of TLR4+ positive cells detected by immunohistochemistry (original magnification ×200). Data are provided as the mean ±SEM (n=6 per group). *means p<0.05 vs CON.


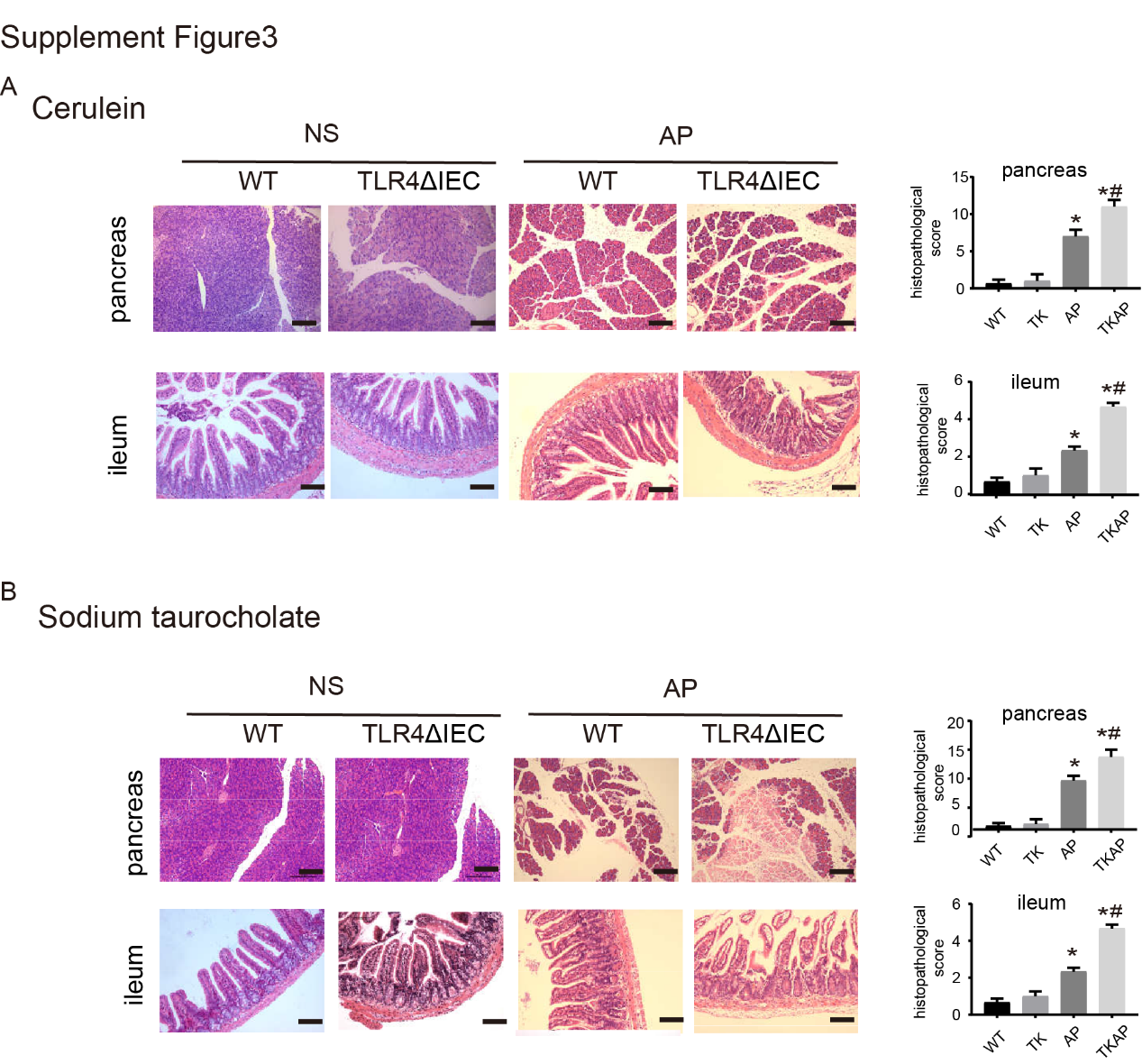


**Supplementary Figure3 The injury of pancreas and ileum in cerulean induced or sodium taurocholate induced AP were aggravated after Intestinal TLR4 silencing**

(a) Representative images of pancreas and ileum stained with HE in cerulean induced AP .Original magnification 100x (the upper figures) or 200x (the lower figures).(b) Representative images of pancreas and ileum stained with HE in sodium taurocholate induced AP. Original magnification 100x (the upper figures) or 200x (the lower figures). Data are provided as the mean ±SEM (n=6 per group). *means p<0.05 vs WT, #means p<0.05 vs AP.


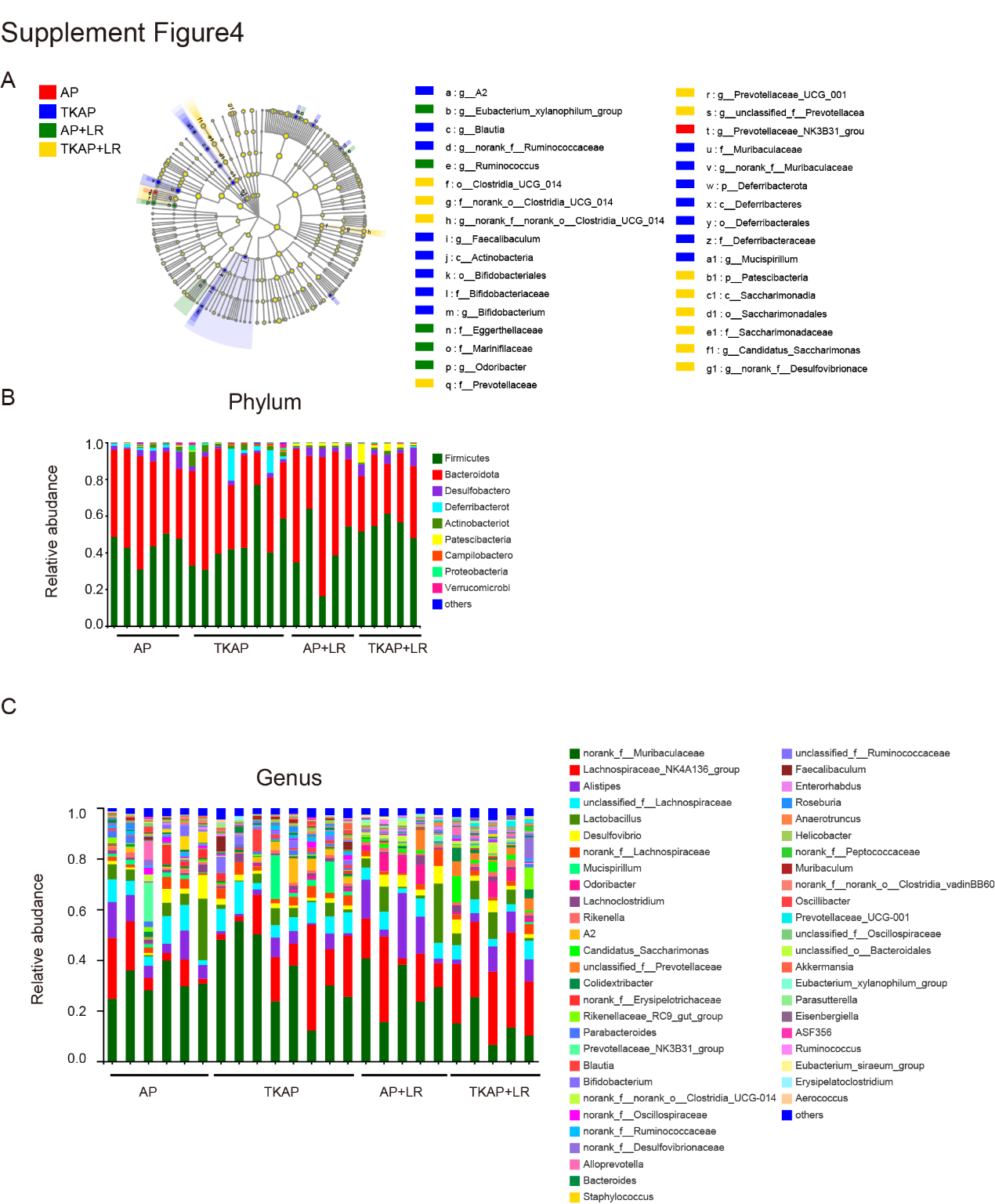


**Supplementary Figure4 Microbiota dysbiosis was improved in mice feed with *Lactobacillus reuteri***

(a) Cladograms generated by LEfSe shows the differences in taxa among four groups(from phylum to genus level). (b) The taxonomic composition distribution among four groups on phylum-level.of fecal microbiota (c) The taxonomic composition distribution among four groups on genus-level of fecal microbiota. (n＞5 per group).

**
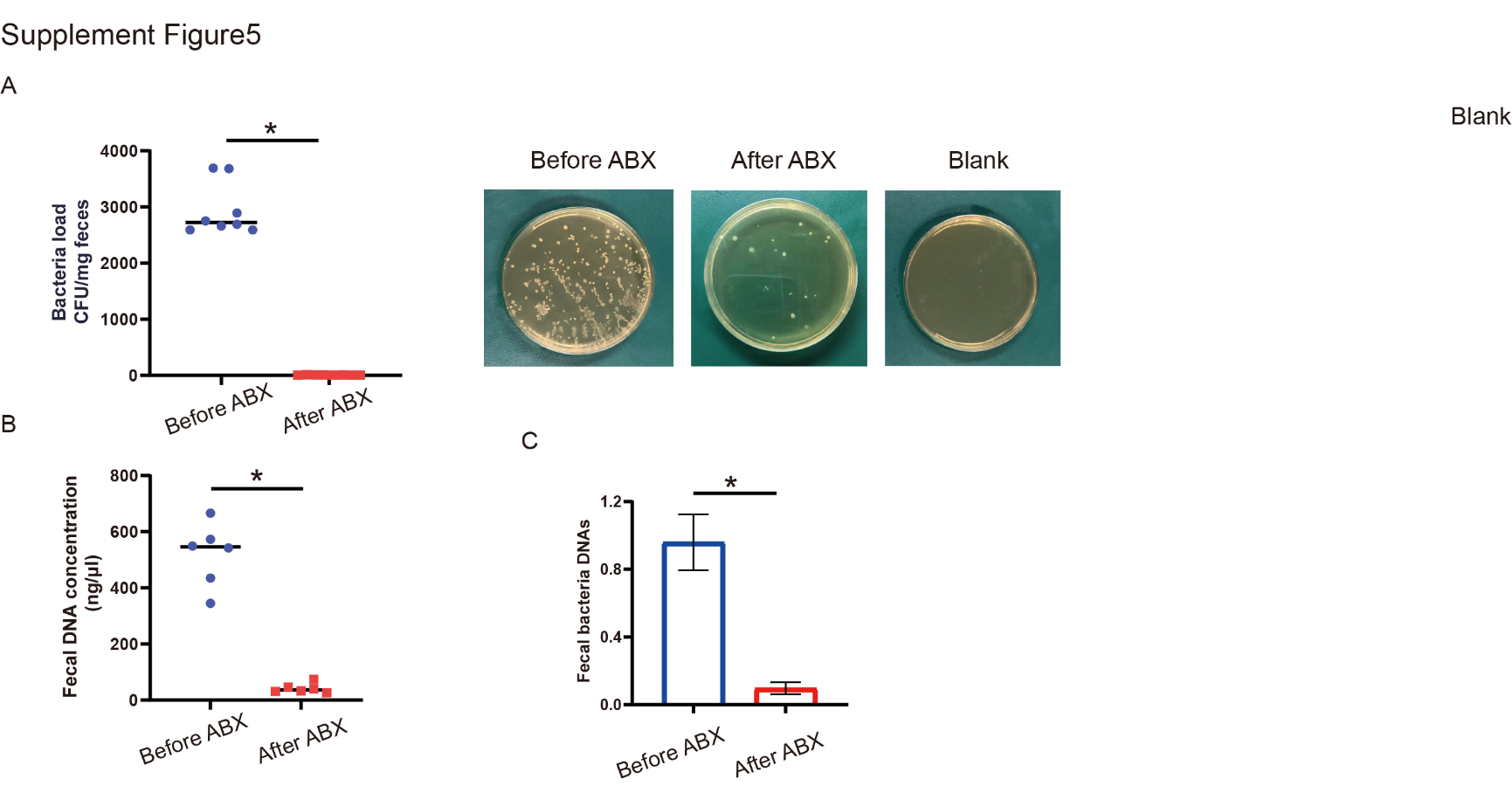
**

**Supplementary Figure5 Antibiotic treatment could effectively deplete the gut microbiota of mice.**

Enteric germ-free mice were generated by drinking with antibiotic cocktail for 4 weeks. Antibiotic water bottles were inverted every day.(a) BHIA plate counting and representative photos of bacterial culture. (b) Concentration of fecal bacterial DNAs before or after antibiotic treatment.. (c) Relative fecal 16S bacterial rDNAs before or after antibiotic treatment. Data are provided as the mean ±SEM (n=6 per group). *means p<0.05.

**
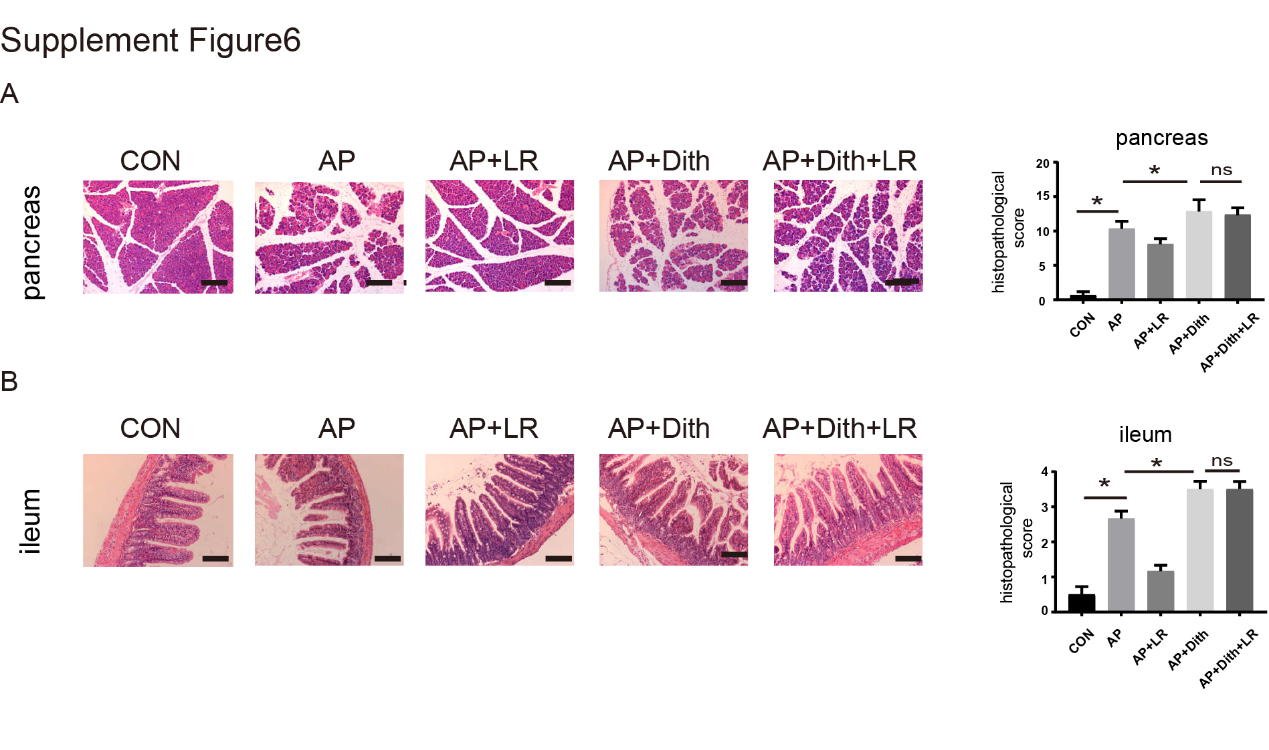
**

**Supplementary Figure6 The injury of pancreas and ileum in AP were aggravated after Paneth deletion and then relieved with *Lactobacillus reuteri* feeding**

Mice were all pretreated with ABX 14 days to eliminate the gut flora. Mice in Dith group were intraperitoneally injected with 40 mg/kg dithizone every three days for two weeks to ablate Paneth cells before AP induction. Mice in LR group were gavaged with *Lactobacillus reuteri*(1*10^8 CFU,2 weeks) before AP induction. Mice in Dith+LR group received both treatments.(a) Representative images of pancreas stained with HE in L-arg induced AP .Original magnification 100x.(b) Representative images of ileum stained with HE in L-arg induced AP. Original magnification 200x. Data are provided as the mean ±SEM (n=6 per group). *means p<0.05. ns means p＞0.05.

**Supplementary Figure7 Graph abstract**


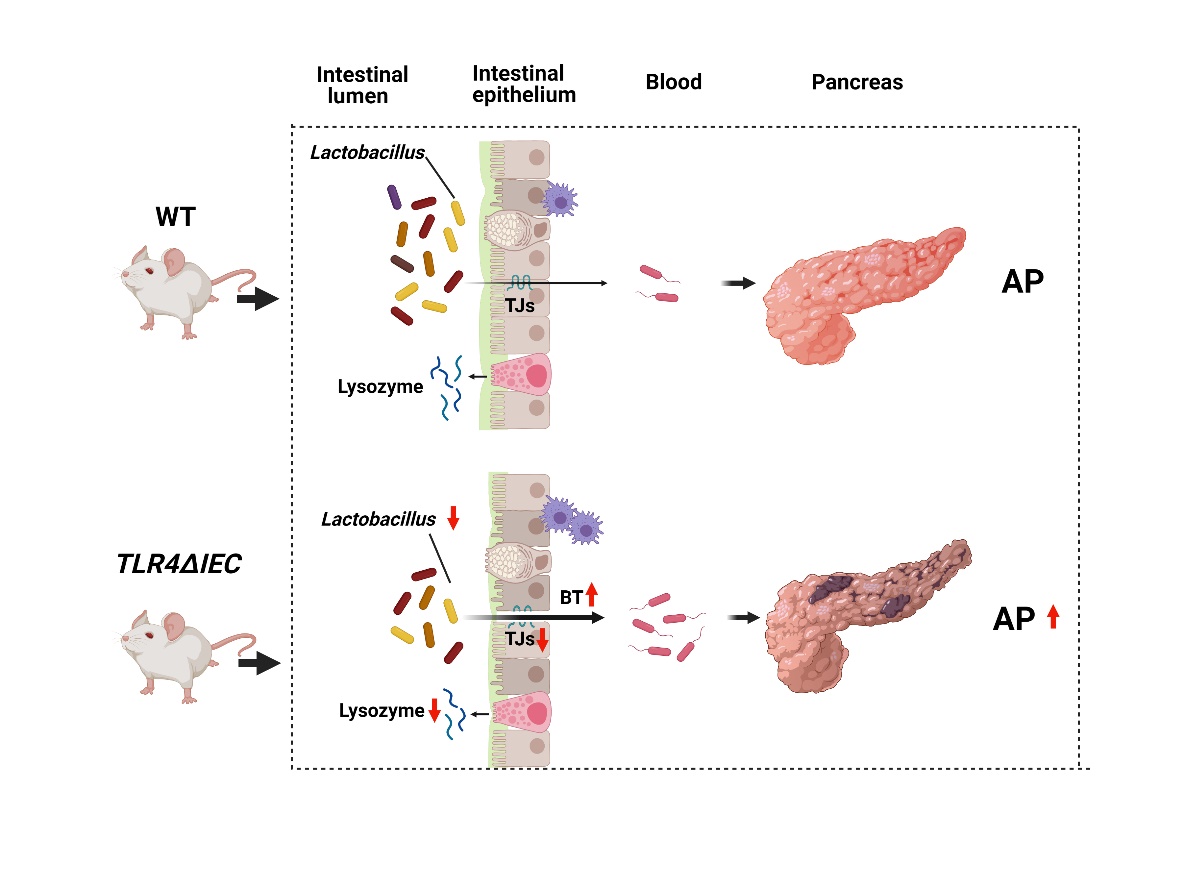


The present study showed that deletion of TLR4 in the intestinal epithelium promotes intestinal and pancreatic injury during AP, which may be attributed to altered gut microbiota(exhaustion of Lactobacillus) and disfunction of Paneth cells. Our study demonstrated that *Lactobacillus reuteri* has the ability to modulate Paneth cells and ISC proliferation to maintain intestinal homeostasis and alleviate AP.

| Table S1. PCR genes primers sequences and annealing temperature | | | |
| --- | --- | --- | --- |
| Gene（mice） | Primer | Sequence (5' - 3') | Annealing Temperature |
| *TLR2* | F | ACAGCAAGGTCTTCCTGGTTCC | 61°C |
|  | R | GCTCCCTTACAGGCTGAGTTCT |  |
| TLR4 | F | GAGGACTGGGTGAGAAATGAG | 60°C |
|  | R | GTAGTGAAGGCAGAGGTGAAAG |  |
| TLR9 | F | GCTGTCAATGGCTCTCAGTTCC | 60°C |
|  | R | CCTGCAACTGTGGTAGCTCACT |  |
| Lysozyme1 | F | TACAACCGTGGAGACCGAAGCA | 62°C |
|  | R | TGGCTGCAGTGATGTCATCCTG |  |
| Defensin alpha 5 | F | CTAATACTGAGGAGCAGCCAGG | 60°C |
|  | R | GCAGCCTCTTATTCTACAATAGCA |  |
| Nod2 | F | CTTCTACAGCACGTCAGGGAAC | 61°C |
|  | R | CTCCAGGCAAAGATTCTCCG |  |
| Lgr5 | F | CTGAGACAGGTTCCGGAGGA | 60°C |
|  | R | GAGATGCAGAACCACGAGGC |  |
| Tublin | F | GGCAGTGTTCGTAGACCTGGAA | 59°C |
|  | R | CTCCTTGCCAATGGTGTAGTGG |  |
