# Supplementary material for: Intestinal TLR4 deletion exacerbates acute pancreatitis through gut microbiota dysbiosis and Paneth cells deficiency
Source: Gut Microbes. 2022 Aug 18;14(1):2112882. doi: 10.1080/19490976.2022.2112882 (PMC9397436; doi:10.1080/19490976.2022.2112882)
Supplement: Supplemental Material [file KGMI_A_2112882_SM2628.zip › revised supplemental materials and methods202208.docx]

**SUPPLEMENTAL MATERIALS AND METHODS**

**Reagents**

Caerulein (HY-A0190) was obtained from MedChem Express (Shanghai, China). Lipopolysaccharide (LPS, #L2880) L-arginine(L-Arg, #A5131), sodium taurocholate(ST,#T4009) Dithizone (Dith, #[43820](https://www.sigmaaldrich.cn/CN/zh/product/sial/43820)) was purchased from Sigma-Aldich Chemical (MO, USA).

**Histopathology and Immunohistochemistry**

After sacrifice, pancreatic and intestinal tissues were fixed in 4% formalin for 24h and then embedded in paraffin, cut into 4 μm sections for hematoxylin and eosin (H&E) staining. Morphological changes of pancreas and ileums were assessed by two pathologists in a blinded manner using light microscope (Leica, Germany). Evaluation of the histopathological changes of pancreas and ileums during AP was in accordance with the scoring scale reported by Schmidt criteria^[1]^ and the Chiu's standard^[2]^. Globet cells was detected by staining PAS by using standard procedures.

**Immunofluorescence**

The antigen of the paraffin embedded section (4 μm thick) was retrieval with boiling citrate buffer (pH 6.0). Endogenous peroxidase activity was blocked by 3% H_2_O_2_ solution at room temperature for 15min. Sections were then incubated with the primary antibody at 4 °C overnight followed by incubation with fluorescein-labeled secondary antibody(1:100,Yeason,Shanghai) for 30 min. The nucleus was visualized by counterstained with DAPI for 10 min. The primary antibody used in this study: Occuldin (1;100, ab216327, Abcam, USA), ZO-1(1;100, ab221547, Abcam, USA), Claudin1(1;100, ab125028, Abcam, USA), PCNA (1;100,A0264, Abclonal, CN)and Lysozyme (1;100,A0099, Dako, Danmark).

**Measurements of serum amylase, lipase, inflammatory cytokines**

Serum concentrations of amylase and lipase were measured by enzymatic kinetic chemistry using commercial kits(Roche, Berlin, Germany)according to the manufacturer's protocol. Serum activities of IL-1β, TNFα, IL-6 were determined using the Luminex Screening Human Magnetic Assay（R&D Systems, MN, USA).

**Bacterial translocation**

Fluorescence in situ hybridization (FISH) was performed to detected Bacterial translocation. Briefly, sections encompassing the distal ileum and pancreas were cut, dewaxed (60 m at 60 °C, 2 × 10 minutes with 100% xylene, 5 minutes with 100% ethanol), air dried, and incubated with specific probes in a humid space at 52 °C for 18 hours. Probes employed in this experiments were as follows: (EUB338: 5' - Cy3 - GCTGCCTCCCGTAGGAGT - 3')16. Then nuclei were counterstained by DAPI for 10min. The confocal microscope (Olympus, Japan)was used to obtain the pictures.

**Tunel staining**

TUNEL assay kit (Roche, 11684817910) was applied to determine the apoptotic cell death in the small. All cell counts were performed at 200x magnification. The apoptotic rate was determined as the average percentage of positive cells in 10 randomly selected areas.

**Intestinal permeability**

Intestinal permeability was measured by quantifying the absorption of FITC-dextran (FD4000; Sigma-Aldrich, MO, USA) after orogastric gavage (0.5 g/kg). The mice were sacrificed 4 h after

gavage , and the blood levels of FITC-dextran were determined as previously described^[3]^.

**Western blotting**

Tissues of pancreas or ileums were homogenized in lysis buffer（RIPA） supplemented with complete protease inhibitor cocktail. BCA protein quantitative assay kit (beyotine biotechnology, China) was used to measure the protein concerntrations. The extracts were separated by SDS-PAGE then transferred to a polyvinylidene fluoride (PVDF) membrane.. After blocking with 5% fat-free milk for 1 hours at room temperature, the membranes were then incubated overnight with primary antibodies at 4 ℃ followed by incubation with secondary antibody combined with HRP. Antibodies against TLR4 were purchased from Cell Signaling Technology(14358) , TLR2 and TLR9 was purchased from Abclonal(A11225 and A14642).The protein bands were observed by Image J software and standardized to tubulin level.

**Real-time PCR**

Total RNA from ielums was extracted by Trizol reagent(Invitrogen, USA) according to the manufacturer’s protocol. Then reverse transcription was performed by using the cDNA cycle kit (Invitrogen, USA). The results were standardized to the control values of 18S and Tublin. Mixture of SYBR green and the ABI 7300 fast real-time PCR system (Applied Biosystems) were used to display Real-time PCR. The methods of ΔΔCT was performed to calculated the relative gene expression. Primers employed in this study are listed in Tables S1.

**Antibiotic treatment**

Enteric germ-free mice were generated by drinking with antibiotic cocktail for 4 weeks. Antibiotic water bottles were inverted every day. The mixture of antibiotic solution applied in this experiment consisted of following antibiotics: ampicillin (1 g/l, Sangon, China), vancomycin (0.5g/l, Sangon, China), neomycin (1g/l, Sangon, China) and metronidazole (1g/l, Sangon, China).

**Detection of bacterial in mice feces**

At the time of collection, mice feces were collected in a sterile tube, homogenized using a high-throughput grinder (Onebio. Biotech, CN), and plated onto brain heart infusion agar (BHIA) plates for culture of anaerobic bacteria. The plates were incubated at 37 ℃ under anaerobic conditions. Then, the counting of colony-forming units (CFUs) was measured after 72 h culturing.

To detect bacterial DNAs in mice feces, DNAs were isolated from approximately 200 mg feces using the E.Z.N.A. Stool DNA Kit (Omega, USA) with the manufacturer’s instructions. The concentration of DNAs was detected by NanoDrop2000 (Thermo Scientific, USA). Real time PCR was performed with QuantStudio 6 Flex Realtime PCR Systems (Thermo Scientific, USA) using universal primers for the bacterial 16S ribosomal RNA gene (Eub forward 5’-ACTCCTACGGGAGGCAGCAG-3’, reverse 5’-ATTACCGCGGCTGCTGG-3’). Fecal bacterial DNA levels were normalized to host 18S rRNA (forward 5’-CTGAGAAACGGCTACCACATC-3’, reverse 5’-GCCTCGAAAGAGTCCTGTATTG-3’) in mice samples.

**Analysis of Gut microbiota**

DNA from the luminal contents of the ileocecum samples of mice was extracted using E.Z.N.A. Stool DNA Kit (Omega, USA) according to the manufacturer’s instructions and amplified using forward (5'-ACTCCTACGGGAGGCAGCAG-3') and reverse(5'- GGACTACHVGGGTWTCTAAT-3'). Then we displayed High‐throughput sequencing and analysis by using Majorbio cloud. (Shanghai, China). The raw sequencing reads of this study are openly available in BioProject at https://www.ncbi.nlm.nih.gov/bioproject/PRJNA824019, referencenumber PRJNA824019.

PCR amplification cycling was performed as follows: initial denaturation at 95℃ for 3 min, followed by 27 cycles of denaturing at 95℃ for 30 s, annealing at 55℃ for 30 s and extension at 72℃ for 45 s, and single extension at 72℃ for 10 min, and end at 4℃. After demultiplexing, the resulting sequences were quality filtered with fastp (0.19.6) and merged with FLASH ( v1.2.11).

Bioinformatic analysis of the gut microbiota was carried out using the Majorbio Cloud platform (https://cloud.majorbio.com).Correlations between the relative abundance of lactobacillus, histopathological score, and serum amylyse, and lipase were calculated using Spearman’s analysis.

**Culture of Lactobacillus reuteri**

Lactobacillus reuteri was cultured as previously described^[4]^.Briefly, Lactobacillus reuteri was provided by Biogaia, Inc. (Stockholm, Sweden) and anaerobic cultured in deMan-Rogosa-Sharpe (MRS; Difco, Detroit, MI) medium at 37°C for 24 h. Then bacteria were plated in MRS agar at specific serial dilutions and grown anaerobically(37°C,48–72 h). A standard curve of bacterial CFU/ml and the absorbance (at 600 nm) of cultures at known concentrations were used to perform quantitative analysis of bacteria. Lactobacillus reuteri in the culture media were harvested by centrifugation (1500 g,15 min) and were resuspended in formula before feeding.

**Intestinal organoid culture**

Enteroids were isolated from C57BL/6 mice and cultured as described previously with the modifications^[5]^ Briefly, the small intestines of mice（8-12weeks）were cut into 2-3mm pieces and washed and then incubated with 2 mM ethylenediaminetetraacetic acid (EDTA) in PBS for 30 min at 4°C on a rotating wheel, and crypts were detached from the basal membrane by vigorous shaking. Crypts enriched in the supernatant were passed through a 70 μm strainer and centrifuged at 800 g.The pelleted crypts were resuspended and seeded in Matrigel (BD Bioscience) on a prewarmed 24-well plate and incubated for 10 min at 37°C. Then, 750 μl of complete crypt culture medium（intestiCult Organoid Growth Medium，stem cell，#06005）was added. Intestinal organoids were cultured at 37°C in a 5% CO2 atmosphere. To explore the interactions of IEC-lactobacillus, enteroids were pretreated with or without L. reuteri (1×106 CFU) Matrigel for 48 h and then treated with TNF (60 ng/ml) for 12 h to induce intestinal damage to the enteroids.

**RNA-sequence**

Total RNA was extracted from the intestines of mice using TRIzol® Reagent (Plant RNA Purification Reagent for plant tissue) according the manufacturer’s instructions (Invitrogen) and genomic DNA was removed using DNase I (TaKara).RNA-seq transcriptome library was prepared by TruSeqTM RNA sample preparation Kit from Illumina(San Diego, CA). RNA-seq library was

sequenced with the Illumina HiSeq xten/NovaSeq 6000 sequencer (2 × 150 bp read length). The raw paired end reads were trimmed and quality controlled by SeqPrep (https://github.com/jstjohn/

SeqPrep) and Sickle (https://github.com/najoshi/sickle) with default parameters. Then clean reads

were separately aligned to reference genome with orientation mode using TopHat (http://tophat.

cbcb.umd.edu/, version2.0.0) software. To identify differential expression genes (DEGs), the expression level of each transcript was calculated according to the fragments per kilobase of exon

per million mapped reads (FRKM) method. GO functional enrichment and KEGG pathway analysis

were carried out by Goatools (https://github.com/tanghaibao/Goatools) and KOBAS (http://kobas.

cbi.pku.edu.cn/home.do). The data were deposited to the GEO database (accession number: GSE200780)

**Reference**

[1] Shimizu T, Shiratori K, Sawada T, et al. Recombinant human interleukin-11 decreases severity of acute necrotizing pancreatitis in mice[J]. Pancreas, 2000, 21(2): 134-40.

[2] Chiu C J, Mcardle A H, Brown R, et al. Intestinal mucosal lesion in low-flow states. I. A morphological, hemodynamic, and metabolic reappraisal[J]. Arch Surg, 1970, 101(4): 478-83.

[3] Cani P D, Possemiers S, Van De Wiele T, et al. Changes in gut microbiota control inflammation in obese mice through a mechanism involving GLP-2-driven improvement of gut permeability[J]. Gut, 2009, 58(8): 1091-103.

[4] He B, Hoang T K, Tian X, et al. Lactobacillus reuteri Reduces the Severity of Experimental Autoimmune Encephalomyelitis in Mice by Modulating Gut Microbiota[J]. Front Immunol, 2019, 10: 385.

[5] Wu H, Xie S, Miao J, et al. Lactobacillus reuteri maintains intestinal epithelial regeneration and repairs damaged intestinal mucosa[J]. Gut Microbes, 2020, 11(4): 997-1014.
